# 肺肿瘤多学科会诊现况与医用大模型赋能下展望

**DOI:** 10.3779/j.issn.1009-3419.2026.106.12

**Published:** 2026-05-20

**Authors:** Yingxin FU, Li SHAN, Tingting YU

**Affiliations:** 830011 乌鲁木齐，新疆医科大学附属肿瘤医院肺内科; Department of Pulmonary Medicine, Affiliated Cancer Hospital of Xinjiang Medical University, Urumqi 830011, China

**Keywords:** 肺肿瘤, 多学科诊疗, 诊疗模式, 大语言模型, 临床决策支持, Lung neoplasms, Multidisciplinary team, Diagnosis and treatment mode, Large language model, Clinical decision support

## Abstract

肺肿瘤为发病率与死亡率最高的恶性肿瘤之一，诊疗复杂，涉及多学科。多学科诊疗（multidisciplinary team, MDT）是现代肺肿瘤诊疗核心模式，通过整合多学科专家智慧，为患者制定个体化最优方案，可显著提升诊疗质量和预后。本文系统梳理了肺肿瘤MDT的组织架构、标准流程、临床价值以及推广中存在的数据整合困难、决策效率不一、资源分配不均等关键问题；重点探讨以人工智能大语言模型为代表的新技术对传统MDT模式的革新作用，分析其在多模态医疗数据的高效融合、实时循证的决策支持、会诊流程与资源优化、精准个体化治疗等方面的应用价值，为构建新一代智能、高效、精准的肺肿瘤MDT平台提供理论依据与发展思路。

## Abstract (en)

Lung cancer ranks among malignant neoplasms with the highest incidence and mortality, and its diagnosis and treatment are complicated, requiring multidisciplinary participation. Multidisciplinary team (MDT) consultation is the core model for modern lung cancer management. By pooling the expertise of specialists from diverse disciplines, MDT develops optimal individualized treatment regimens for patients and markedly improves diagnostic and therapeutic quality as well as patient prognosis. This article systematically summarizes the organizational framework, standardized procedures and clinical value of lung cancer MDT, alongside key problems in its popularization including obstacles in data integration, inconsistent decision-making efficiency and imbalanced resource allocation. It elaborates on the innovative effect of new technologies represented by artificial intelligence large language models on conventional MDT modes, and analyzes their application value in high-efficiency integration of multimodal medical data, real-time evidence-based decision-making support, optimization of consultation procedures and resources, as well as precise individualized treatment, so as to furnish theoretical basis and development ideas for establishing a new-generation intelligent, efficient and precise lung cancer MDT platform.

肺肿瘤异质性强，发病率与死亡率居恶性肿瘤首位^[1]^，其治疗策略复杂，涉及胸外科、肿瘤内科、放疗科、影像科、病理科等多学科决策^[2]^。传统单学科模式难以满足个体化治疗需求，以患者为中心的多学科诊疗（multidisciplinary team, MDT）已成为肺肿瘤诊疗标准模式^[3]^。MDT通过整合多学科专家经验制定最优方案，可显著改善患者生存与生活质量^[4]^，尤其在III期非小细胞肺癌（non-small cell lung cancer, NSCLC）的可切除性评估与综合治疗决策中至关重要^[5]^。但当前MDT仍面临多重挑战：专家时间协调困难，影响会诊及时性与常态化^[3,6]^；影像、病理、分子检测及临床病史等多源数据整合效率低下，制约精准决策^[7]^；诊疗过度依赖专家经验，缺乏标准化数据驱动工具，易导致决策差异^[8]^；医疗资源分布不均使基层难以开展高质量MDT，加剧诊疗不公^[3,6]^，严重限制其临床价值。

近年来医用人工智能（artificial intelligence, AI）大模型快速发展，为突破上述瓶颈、重构MDT流程提供了新路径^[7]^。其在自然语言处理、多模态融合与复杂推理方面优势突出。在第26届世界肺肿瘤大会上杨懿教授团队的肺肿瘤MDT专用孔明AI系统，可整合循证知识库与影像分析，实现流程自动化和会诊决策支持，提升会诊效率；清华MedMPT作为呼吸专科多模态预训练模型，可融合电子计算机断层扫描（computed tomography, CT）影像、报告、结构化数据与用药知识，为MDT前端数据整合提供支撑^[9]^；腾讯DeepGEM仅需常规病理切片即可1 min内预测肺肿瘤驱动基因突变，准确率为78%-99%，较下一代测序（next-generation sequencing, NGS）成本降低70%以上，时间由7-14天缩至1 min^[10]^，展现出临床辅助决策价值。由此可见，医疗大语言模型可从诊疗效率、诊断精准度、服务可及性3个维度赋能MDT：通过自动病例归纳与分诊提升效率；依托循证医学证据输出优化诊疗精准度；借助远程协作与知识下沉提升基层服务能力，进而推动以患者为中心、全周期个体化诊疗的落地。

## 1 肺肿瘤MDT的现况与核心要素

### 1.1 组织架构与标准化流程

规范化肺肿瘤MDT核心团队由胸外科、肿瘤内科、放疗科、影像科、病理科副主任及以上专家构成^[11]^。针对局部晚期NSCLC等复杂疑难病例，需扩充纳入呼吸内科、介入科、核医学等亚专科专家，从肿瘤侵犯范围、手术可切除性、分子特征等维度开展综合评估^[12]^。标准化运行流程是保障MDT质量与效率的基石。这一流程通常始于病例的筛选与准备，由专门的协调员负责整理包括影像学、病理学及分子检测报告在内的所有关键资料。随后MDT团队固定时间、固定地点开展会诊（支持线上参会），各学科专家依次陈述观点，经过充分的集体讨论后形成共识性的诊疗建议^[11,13]^。有丹麦学者^[14]^通过向全国4家肺肿瘤MDT中心发放模拟病例评估发现，不同中心在临床分期与治疗策略选择中存在明显异质性，凸显标准化会诊流程对统一临床判定标准的重要价值。MDT形成的诊疗意见需完整记录并严格执行，系统追踪患者依从性与远期预后，完善的MDT病例登记库、定期回顾复盘与预后监测是成熟MDT质量管控体系的核心组成^[15]^。

### 1.2 临床价值与循证证据

肺肿瘤MDT的临床价值已得到大量循证医学佐证，主要体现在规范诊疗决策、改善患者远期预后、加速前沿技术临床转化三方面。MDT整合多学科视角，严格遵循最新指南与循证依据制定方案，可有效规避治疗不足与过度医疗^[11]^。研究^[16]^证实，MDT可提升疾病诊断与分期精准度，优化治疗方案；III期NSCLC经MDT会诊患者中位生存期显著优于单科诊疗人群，凸显个体化决策核心价值。MDT是开展临床试验、推动前沿治疗技术临床转化的重要平台。精准医疗时代下，MDT可整合靶向治疗、免疫治疗最新研究进展，制定个体化诊疗策略。研究^[17]^表明，随着精准肿瘤学的不断发展，规范MDT支撑下的个体化治疗不仅可延长局部晚期NSCLC患者生存期，还能加速前沿研究成果落地临床，使患者及时受益于医学新进展。

### 1.3 面临的主要挑战与瓶颈

尽管MDT临床价值明确，但国内规模化落地仍存在诸多瓶颈，首要问题是医疗资源分布失衡。国家癌症中心《2025年全国肺癌诊疗质量控制报告》显示，国内70%以上的疑难肺肿瘤病例由三级医院承接，但优质资源高度集中于中心城市，放疗、病理专科医师缺口显著。目前仅35%的三级医院可开展全学科规范化MDT，县域医院MDT覆盖率仅25%，多数基层团队仅能完成内科联合影像的基础协作，无法满足精准诊疗需求^[6]^。基层分子诊断技术覆盖率不足23%，而指南明确要求晚期NSCLC常规开展表皮生长因子受体（epidermal growth factor receptor, EGFR）、间变性淋巴瘤激酶（anaplastic lymphoma kinase, ALK）、c-ROS原癌基因1-受体酪氨酸激酶（c-ROS proto-oncogene 1 receptor tyrosine kinase, ROS1）等驱动基因检测，技术短板使基层MDT难以制定符合指南的靶向方案，患者只能大量向上转诊。其次，《医疗保障按病种付费管理暂行办法》表示现行医保支付体系未将MDT纳入核心付费目录，仅部分地区按补充项目收费，导致院方与医师动力不足。单例MDT人力成本为300-500元，而多地医保定价仅150元，医院开展即亏损，医师劳务报酬偏低，参与积极性不高；并且，按项目付费与MDT全病程管理模式冲突，支付多限于住院阶段，门诊、随访及康复期MDT难以报销，无法形成全周期管理，价值难以充分发挥。此外，国内公立医院以科室为绩效单元，考核与资源分配均以科室为单位，与MDT跨学科协作存在天然矛盾，仅3%-8%的医院将MDT参与度纳入科室核心考核且权重偏低，学科话语权不均、利益分配缺失致使约37%的MDT讨论难以形成可行方案，会诊易流于形式，方案多由强势科室主导^[6]^；同时，跨科室数据调用流程繁琐、审批慢，约30%基层转诊患者本可就地处置，却因数据与协作障碍被迫转诊，加剧资源浪费与患者负担^[6]^。

## 2 医用大模型的技术特征及其在医疗领域的应用基础

### 2.1 大模型的核心能力：从自然语言理解到多模态融合

首先，医用大模型依托海量医学文献、指南及真实世界文本预训练，具备强大上下文理解与指令跟随能力，可自动梳理结构化病历、提炼病情要点、生成鉴别诊断思路，显著提升临床文书规范性与书写效率^[7]^。在儿科肿瘤等领域，大模型辅助生成的手术文书关键信息完整度显著优于传统人工书写，可为MDT讨论提供标准化信息支撑^[18]^。其次，新一代医用大模型已迈入多模态融合阶段，可同步解析CT、病理切片、实验室数据、基因组学、临床病历等多维度信息，构建患者全息诊疗画像^[7,9]^。以清华MedMPT为代表的多模态模型，可自动识别肺部影像病灶、量化形态特征，实现影像与文本数据深度关联，为MDT精准评估提供多维依据^[7,9]^。最后，通过在海量医学文献、教科书、指南共识和真实世界数据上进行预训练与精调，这些模型内嵌了庞大的医学知识网络，能够进行一定程度的医学逻辑推理和证据链追溯^[7]^。这种能力使得模型能够快速关联患者的特定表现与最新的循证医学知识，以肺肿瘤MDT为例，模型可即时检索、归纳并推送与III期NSCLC病例有关的最新临床试验与指南推荐，为MDT团队提供实时决策支撑^[19]^。

### 2.2 在辅助诊断与临床决策中的初步实践

医用大模型已在临床辅助诊断与决策场景中展现良好应用潜力。大语言模型可通过智能人机交互自动生成结构化病历，大幅降低医师文书负担、提升工作效率^[7]^。多模态大模型可精准完成肺结节检出、良恶性鉴别及病灶特征量化，结构化输出CT影像关键信息，为MDT精准评估提供客观依据^[7,9]^。同时，大模型具备高效的文献与指南更新能力，可针对个体化病例快速梳理前沿研究与指南共识，为临床决策提供实时循证支撑^[7]^。初步研究^[20]^显示，大型语言模型在基于早期乳腺癌患者病历生成辅助治疗建议时，与MDT专家决策吻合度较高，尤其在辅助内分泌治疗和靶向治疗的建议上表现出色。然而，当前模型在涉及复杂手术决策或特定基因组学检测指征时仍存在局限性，表明其更适合作为增强人类决策的工具，而非替代^[20,21]^。

## 3 医用大模型赋能肺肿瘤MDT的变革路径

### 3.1 会诊前：智能化病例准备与数据整合

大模型接入医院信息系统后，自动解析电子病历、影像报告、病理报告、基因检测、影像组学、既往治疗史，一键生成结构化MDT病例摘要^[3]^。依托多模态融合与可视化能力，模型可对III期NSCLC患者的CT肿瘤特征、免疫组化结果、基因突变信息开展关联分析并可视化展示，帮助专家快速判定肿瘤侵犯范围、淋巴结转移状态等可切除性核心指标^[9,10,22]^。同时，模型可结合患者个体化数据与最新循证指南，预生成鉴别诊断、分期复核、备选治疗方案、潜在风险及待完善检查清单。针对局部晚期NSCLC，可同步推荐手术综合治疗、根治性放化疗、放化疗后免疫巩固等个体化方案，并匹配对应临床研究证据，为MDT研讨提供标准化决策框架^[19,23]^。

### 3.2 会诊中：实时决策支持与知识增强

在会诊进行过程中医用大模型能够扮演“虚拟专家”的角色，提供实时的知识增强与决策支持。当专家讨论到具体细节时，例如“III期EGFR 19del伴脑转移，优先方案与证据?”“奥希替尼+放疗的安全性数据?”“免疫单药 vs 联合治疗的生存期对比?”等问题时，专家可随时向模型提问并即时获得基于指南条文、文献摘要、相似病例方案^[7]^。这种能力极大地扩展了MDT的知识边界，确保决策基于最前沿最真实的证据。当专家意见出现分歧时，大模型的量化分析功能显得尤为重要。例如，在判断一个侵犯纵隔结构的T4期肿瘤是否可切除时，不同专科专家可能基于自身经验有不同看法^[22]^。此时，模型可调用已有的相似病例大数据，模拟不同治疗方案（如直接手术、新辅助治疗后手术或根治性放化疗）下的预期疗效与风险，提供概率化的预后预测，从而辅助团队达成共识^[24]^。此外，大模型还能实时转录并结构化记录整个讨论过程。它能够自动识别并提取关键信息，如最终确定的诊疗计划、具体的治疗细节分配以及详细的随访计划，生成规范化的会诊记录^[7]^。这不仅减轻了人工记录的负担，也确保了治疗计划的准确传达与执行。

### 3.3 会诊后：方案执行跟踪与疗效预测

大模型的赋能价值贯穿会诊后全流程，可实现方案宣教、疗效监测、质量迭代的闭环管理。根据MDT确定的个性化治疗方案，大模型可自动生成通俗易懂的患者教育材料。这些材料能详细说明治疗步骤、潜在副作用、注意事项及康复建议，从而提高患者的治疗依从性和自我管理能力^[25]^。大模型还能够整合治疗开始后的动态数据，实现疗效预测与早期预警。通过持续分析循环肿瘤DNA（circulating tumor DNA, ctDNA）、定期影像复查（如CT）等数据，模型可以动态评估治疗反应，早期预警耐药或疾病进展的风险^[26]^。例如，对于接受放化疗后免疫巩固治疗的不可切除III期NSCLC患者，若模型预测到早期进展的高风险，可及时提示临床团队，考虑是否需要再次启动MDT讨论以调整治疗策略^[19]^。最终，所有经由大模型辅助的MDT决策过程与结果均可被系统化地记录、存储与分析，从而构建一个高质量、可追溯、可审计的决策知识库^[7]^。这个知识库具有多重价值：一方面，其中的真实世界案例可用于培训年轻医生，展示复杂情况下的多学科决策逻辑；另一方面，通过对大量决策案例与最终结局的分析，可以用于MDT质量控制，评估不同决策路径的长期效果，并反过来迭代和优化大模型自身的算法，形成一个“临床实践-数据沉淀-算法升级”的持续改进闭环^[27]^。

## 4 未来展望、挑战与实施路径

### 4.1 发展前景：构建下一代智能MDT生态系统

下一代智能MDT生态的核心是AI技术与临床工作流的深度融合，破解传统模式效率不足、资源不均的痛点。云端协同智能MDT平台是核心发展方向，可整合多中心标准化病例数据，依托AI完成结构化分析，精准匹配远程专家资源，推动优质诊疗普惠下沉^[7]^。已有集成大语言模型与计算机视觉的智能平台，通过流程自动化与智能决策模块，有效提升会诊效率与数据利用率，为区域智能MDT中心建设提供了实践范式^[7]^。同时，智能MDT生态系统有望推动动态适应性治疗规划。通过将大模型与物联网、可穿戴设备采集的实时生理数据相结合，系统能够持续监测患者对治疗的反应，为治疗方案的动态调整提供依据，从而迈向更精准的个体化管理^[26]^。此外，该平台可依托真实世界数据快速筛选临床试验入组病例、挖掘科研方向，加速新药与新技术研发转化。真实世界研究^[24]^已证实，数据驱动的MDT个体化策略可显著优化局部晚期NSCLC患者生存预后，凸显智能决策的临床核心价值。

### 4.2 面临的伦理、法律与技术挑战

尽管前景广阔，智能MDT的落地应用仍面临数据安全、伦理规范、算法性能与临床适配等多重挑战：（1）数据隐私风险突出，智能平台需处理大量患者敏感信息，需严格恪守数据安全法规，防范信息泄露^[26]^；（2）模型责任与透明度问题亟待厘清，在临床决策中责任主体必须始终是医生，大模型应定位为辅助工具，然而，当前许多先进模型的“黑箱”特性使其推理过程难以理解，这要求我们向“可解释AI”发展，使其决策依据对临床医生透明以建立信任^[21]^；（3）算法偏见与泛化能力是影响模型公平性与有效性的核心问题，如果训练数据存在偏差（如某些人群或疾病亚型的数据不足），模型可能对特定群体产生不公甚至有害的决策建议^[28]^，需依托多源异构数据集完成训练验证，保障模型稳定性与公平性；（4）临床整合与医生接受度是决定技术能否落地的最终环节，这要求设计出符合临床工作流、用户体验良好的人机交互界面，并通过严谨的前瞻性临床研究证明智能MDT工具能切实改善患者长期预后，才能获得广泛采纳^[3]^。一项关于头颈肿瘤的研究^[21]^也指出，尽管大型语言模型能提供可行的治疗建议，但目前它们应作为人类决策的补充而非替代，强调了临床整合中“人在环路”模式的重要性。

### 4.3 可行的实施路径与建议

为了克服挑战并实现智能MDT的平稳落地，需要制定审慎而可行的实施路径。首先，应采取分阶段推进的策略。初期可以从非核心的辅助任务入手，例如利用AI自动生成结构化病历报告、进行高效的文献检索或整理患者资料，这已在儿科肿瘤等领域显示出提升文档完整性和多学科沟通效率的潜力^[18]^。在积累信任和验证可靠性后，再逐步过渡到辅助影像解读、治疗方案推荐等核心决策支持环节，并始终坚持医生监督下的“人在环路”模式^[9,10,21]^。其次，建立多中心验证联盟至关重要。医疗机构、高等院校和AI公司应联合开展大规模、前瞻性的临床验证研究，以评估智能MDT对患者长期生存、生活质量和医疗成本等重点指标的实际影响^[24]^。第三，强化复合型人才培养，培育兼具临床诊疗能力与AI认知的专业人才，打通技术创新与临床应用的衔接壁垒^[29]^。最后，完善有关的政策与标准是营造健康创新环境的保障。监管机构需尽快制定关于医用AI在辅助决策中应用的审批流程、持续监管框架以及合理的付费标准，为技术的安全、有效和可持续应用提供明确指引^[3]^。

## 5 智能MDT中国特色肺肿瘤诊疗体系建设的启示

### 5.1 坚持“自上而下”的政策引导与“自下而上”的技术探索协同

构建中国特色肺肿瘤诊疗体系，首先要坚持“政策引导+技术探索”的协同——政策引导明确方向，技术探索提供路径，二者缺一不可。从政策层面看，2025年国家卫健委等6部门发布的《关于促进和规范“人工智能+医疗卫生”应用发展的实施意见》，明确了两大核心目标：2027年建立一批AI医疗示范基地和2030年基层诊疗智能辅助应用基本实现全覆盖，这为AI+MDT的发展提供了明确的政策导向。但政策的落地需要“自下而上”的技术探索支撑。头部医疗机构的AI+MDT实践为政策落地提供了可复制的路径，例如陆军军医大学第一附属医院的AI-MDT门诊为基层MDT的推广提供了“技术下沉”的模式；同济大学附属上海市肺科医院的“1+2+N”质控架构为MDT的质控管理提供了“标准化”的模式；广东省人民医院的全流程AI辅助为MDT的效率提升提供了“工具化”的模式。这些实践不仅验证了AI+MDT的可行性，更为政策的细化提供了真实世界的数据支撑。

### 5.2 构建“1+N”的分级诊疗与AI-MDT协同模式

针对我国医疗资源“倒金字塔”分布难题，可构建“1+N”AI-MDT分级诊疗体系：以国家/省级区域医疗中心为核心，承担AI模型训练与质控；基层医疗机构为应用终端，实现优质MDT资源规模化下沉。四川大学华西医院AI-MDT平台覆盖81家联盟医院，基层MDT开展率由12%升至68%，诊疗一致性达82%。截至2025年12月全国80%县（市、区）已建成影像、心电、检验共享中心，为该体系提供了关键技术支撑。“1+N”模式的核心价值在于推动医疗资源均等化，打破地域与层级壁垒，使基层患者可就近获得同质化MDT服务。

### 5.3 推进“智防-智筛-智诊-智治-智康”的全链条整合

构建中国特色肺肿瘤诊疗体系，核心是打破碎片化诊疗模式，实现筛查-诊断-治疗-随访全链条闭环管理，这是提高肺肿瘤生存率的关键。医用大模型可实现“智防-智筛-智诊-智治-智康”全周期数据整合与智能覆盖。四川大学华西医院联合相关机构推出的“华保千结”肺结节全程管理数字化疗法，依托AI-MDT实现风险评估、方案制定与智能随访，将早期肺肿瘤5年生存率从70%提升至85%。全链条模式以患者为中心，实现肺肿瘤诊疗从片段式治疗向全生命周期管理的升级，是肿瘤防治赢在整合的具体表现，成为中国特色诊疗体系的重要特征。

### 5.4 探索可持续的医保支付机制，支撑体系化落地

构建中国特色肺肿瘤诊疗体系需建立可持续的医保支付机制，使支付与MDT价值相匹配，保障体系长效运行。2025年国家医保局相关办法明确，将需MDT的病例纳入疾病诊断相关分组（diagnosis related groups, DRG）、病种分值付费（diagnosis-intervention packet, DIP）高权重病组（≥2.0），提高报销额度以激励医院开展MDT。地方已形成可复制模式，如湖南以多级财政补贴推动AI基层应用，浙江试点AI 辅助诊疗打包付费，推动医保由按项目付费转向按价值付费。以价值为核心的支付机制从重服务数量转向重服务质量与效果，是体系落地运行的关键支撑。

## 6 小结与展望

当前肺肿瘤MDT正处于技术融合与范式转型关键期，传统模式高度依赖专家与优质资源，诊疗质量和可及性受限。医用大模型通过多模态数据与循证知识融合，推动MDT从经验决策走向数据增强决策，在提升精准与效率的同时，助力优质资源均质化下沉。构建智能MDT中国特色肺肿瘤诊疗体系，需坚持4点路径：（1）政策引导与技术探索双向协同；（2）建立“1+N”分级诊疗模式，实现优质MDT资源下沉；（3）打造“智防-智筛-智诊-智治-智康”全链条闭环管理；（4）完善价值导向的医保支付机制，保障体系长效运行。大模型虽优势显著，但存在数据依赖、决策黑箱等局限，未来应走人机协同路线，而非替代专家。最终需医、工、研、产、监多方协作，以临床需求为核心，严守伦理与安全底线，让AI真正赋能肺肿瘤MDT，实现精准、高效、普惠的诊疗升级，从而更好地为人民服务。
